# Inference of differentiation time for single cell transcriptomes using cell population reference data

**DOI:** 10.1038/s41467-017-01860-2

**Published:** 2017-11-30

**Authors:** Na Sun, Xiaoming Yu, Fang Li, Denghui Liu, Shengbao Suo, Weiyang Chen, Shirui Chen, Lu Song, Christopher D. Green, Joseph McDermott, Qin Shen, Naihe Jing, Jing-Dong J. Han

**Affiliations:** 1 Chinese Academy of Sciences Key Laboratory of Computational Biology, Chinese Academy of Sciences-Max Planck Partner Institute for Computational Biology, CAS Center for Excellence in Molecular Cell Science, Collaborative Innovation Center for Genetics and Developmental Biology, Shanghai Institutes for Biological Sciences, Chinese Academy of Sciences, Shanghai, 200031 China; 2 Tsinghua-Peking Center for Life Sciences, Tsinghua University, School of Medicine, Tsinghua University, Beijing, 100084 China; 3 State Key Laboratory of Cell Biology, Institute of Biochemistry and Cell Biology, Shanghai Institutes for Biological Sciences, Chinese Academy of Sciences, Shanghai, 200031 China

## Abstract

Single-cell RNA sequencing (scRNA-seq) is a powerful method for dissecting intercellular heterogeneity during development. Conventional trajectory analysis provides only a pseudotime of development, and often discards cell-cycle events as confounding factors. Here using matched cell population RNA-seq (cpRNA-seq) as a reference, we developed an “iCpSc” package for integrative analysis of cpRNA-seq and scRNA-seq data. By generating a computational model for reference “biological differentiation time” using cell population data and applying it to single-cell data, we unbiasedly associated cell-cycle checkpoints to the internal molecular timer of single cells. Through inferring a network flow from cpRNA-seq to scRNA-seq data, we predicted a role of M phase in controlling the speed of neural differentiation of mouse embryonic stem cells, and validated it through gene knockout (KO) experiments. By linking temporally matched cpRNA-seq and scRNA-seq data, our approach provides an effective and unbiased approach for identifying developmental trajectory and timing-related regulatory events.

## Abstract (web summary)

Single cell transcriptome data can be used to determine developmental lineage trajectories. Here the authors map single cell transcriptomes onto a differentiation trajectory defined by cell population transcriptomes and show that cell cycle regulators have a role in differentiation timing.

## Introduction

Single-cell RNA sequencing (scRNA-seq) technology is a powerful method for analyzing intercellular heterogeneity during development and reprogramming. A key aim of examining such heterogeneity is to discover unknown cellular states or developmental lineage trajectories. Many methods have been developed to reconstruct a developmental pseudotime trajectory based on scRNA-seq inter-cell expression distance alone, such as Monocle^1^ and Wanderlust^2^. Such approaches are quite subject to confounding factors, biological and non-biological^3^. One confounding factor is the cell cycle^4^. A method to remove cell-cycle effects, called “latent variable model (scLVM),” was developed and renders cell-cycle-independent gene expression^4^. However, in some cases—particularly during differentiation—the cell cycle is not only an integral part of the process studied but may also play a regulatory role, e.g., the length of G1 and M phases has been shown to directly affect lineage determination^5–7^. Therefore, to assess the contribution cell-cycle-associated gene expression to a development trajectory, unbiased methods need to be developed. Here we propose an approach to solve this problem by including cell population RNA-seq (cpRNA-seq) data in parallel to the scRNA-seq data as a reference, and then order the single-cell trajectories not based on their inter-cell expression distance, but instead on the external reference time (actual time) derived from the cpRNA-seq data. We applied our method to the in vitro neural differentiation process of mouse embryonic stem cells (mESCs), and show that it can more effectively align the single-cell differentiation trajectories than routine single-cell distance based on pseudotime reconstruction methods. Importantly, as the reference time is the actual time of the differentiation, the predicted time is no longer a pseudotime, but time with an actual time scale. Moreover, co-analysis of cpRNA-seq together with scRNA-seq data allows further identification of upstream regulatory events that give rise to cell heterogeneity, whereas scRNA-seq data alone is unable to. We assembled our computational methods into a downloadable package “iCpSc” (integrate_cpRNA-seq_scRNA-seq), and use mESC neural differentiation as an example to demonstrate the utility of our approach.

Given its great therapeutic potential for various neural degenerative diseases, the directed neural differentiation of pluripotent cells has been under intense investigation. Previous studies have demonstrated that neural development is a step-wise process during in vitro mouse embryonic development, transitioning through the inner cell mass, pluripotent epiblast, late epiblast, neuroectoderm, and mature neuron stages^8–11^. Culturing ESCs in vitro with minimal exogenous signals can mimic the step-wise in vitro neural differentiation and reach differentiation efficiency as high as 80%^12, 13^. Recent cellular and molecular studies have uncovered many molecules and signaling pathways participating in neural commitment. However, how these regulators and other unidentified components act together to regulate early neural commitment is still poorly understood. More importantly, as the differentiation process is rather self-driven after serum withdrawal, it is completely unknown how it is timed at the population and single-cell levels and whether single cells display heterogeneity or synchronization during this process.

Here, we used cpRNA-seq to identify major stages during this process. Then, based on these stages, we selected eight timepoints (two timepoints per stage) to perform scRNA-seq on eight cells for each timepoint to examine the intercellular heterogeneity at each stage. We show that the number of scRNA-seq samples that are sufficient to capture nearly all intercellular heterogeneity of any stage can be determined using the “iCpSc.samplingSaturation” utility in our iCpSc package. Then, by developing the “iCpSc.CpToScTime” utility, we first inferred a linear model for differentiation time using the cpRNA-seq data, and applied this model to the scRNA-seq data to estimate the differentiation time of each single cell. We further demonstrated the utility of the iCpSc package on two other differentiation time course datasets with matching cpRNA-seq and scRNA-seq, including one with branching trajectories. Based on the model-derived time of single cells we identified the genes that show correlated expression with a single cells’ differentiation time (“timer” genes). Surprisingly, we found cell-cycle regulators are involved in timing the differentiation progress of a cell. To enable the achievement of one key aim of scRNA-seq analysis—to infer regulatory networks for cell heterogeneity^3, 14^—we added an “iCpSc.eResponseNet” utility to infer regulatory network flow from cpRNA-seq and scRNA-seq. In mESC neural differentiation we inferred that regulatory genes, such as *Smad1*, *Fyn*, and *Trp53*, have roles as hub genes that coordinate cell-cycle progression and neural commitment at the single-cell level—even though these genes are not detected or lowly expressed in the scRNA-seq data. Finally, by generating a CRISPR/Cas9 KO of *Fyn* or perturbing mESCs with a small molecule inhibitor that promotes M phase, we experimentally validated the role of Fyn and M phase in controlling differentiation timing.

## Results

### Bulk transcriptome changes of neural commitment

After confirming the efficiency of our in vitro differentiation system (Supplementary Fig. 1 and Supplementary Table 1), we selected 14 timepoints based on the expression profiles of marker genes to perform cpRNA-seq analysis, which can best represent different gene expression stages and sub-stages, using 10^6^–10^7^ cells per sample (time points in red font, Supplementary Fig. 1c). Correlation analysis showed that normalized RNA-seq tag counts (reads per kilobase per million (RPKM)) of the markers are highly correlated with their expression levels determined by real-time quantitative polymerase chain reaction (qPCR) (Supplementary Fig. 1f).

**Fig. 1:**
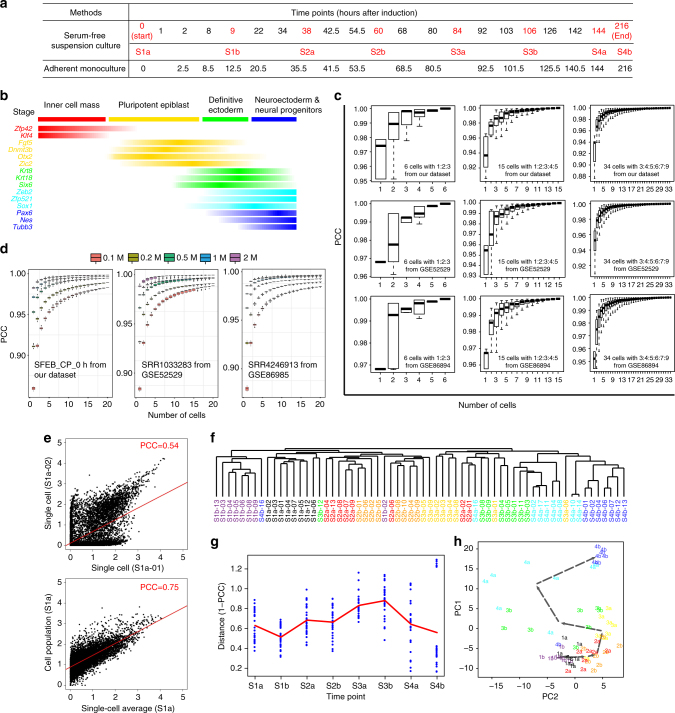
Temporal stages of mESC neural differentiation and intercellular heterogeneity. **a** Time points selected for RNA-seq using two different neuron differentiation methods. The red points are selected for single cell RNA-seq. The “Sxy” pattern is used to label different time points for single-cell RNA-seq, with x representing the stage and y the first **a** or second **b** time point within the stage. Stages were defined by marker expression in **a** (see text for details) and corresponding patterns in cell population RNA-seq.** b** Expression patterns of each stage’s marker genes and representative neural differentiation marker genes as revealed by RNA-seq. **c** Saturation curves using simulated cpRNA-seq from different ratios of single-cell RNA-seq data as reference. Single cells are randomly sampled at the indicated coverage (number of cells) and their profile similarities are compared to the simulated cpRNA-seq data generated from 6 cells with 1:2:3 ratio (left), 15 cells with 1:2:3:4:5 ratio (middle), and 34 cells with 3:4:5:6:7:9 ratio (right) using our mESC dataset (top) or two published datasets (middle and bottom). See Methods for details. **d** Saturation curve after sampling reads at different assumed sequencing depth from 0.1 to 2 million reads (based on sample SRR1033283 from GSE52529, sample SRR4246913 from GSE86985, and sample SFEB_CP_0 h from our dataset, respectively. See Methods for details. **e** Correlations of gene expression levels (*x*- and *y*-axis: log-scale RPKM) between two single cells (left, S1a-01 and Sa1-02), and between the average of 8 single-cell profiles and their corresponding cell population profile (right) across the 11,449 expressed genes (RPKM > 0.5 in at least eight samples). **f** Unsupervised clustering of single cells based on their expression correlation (measured by PCC) across the 11,449 expressed genes. **g** The single-cell pairwise differences (1-PCC) in expression patterns during mESC neural differentiation. **h** Principal component analysis (PCA) of the single cell transcriptomes based on cpDEGs. Eight cells from the same time point are shown with the same color, with the black dotted line connecting the position of average PC1 and PC2 loading of all eight to visualize the temporal progression trajectory

The genes differentially expressed at different time points in the cell population transcriptome data (cpDEGs, see Methods section) were automatically grouped into 16 gene clusters and 4 sample clusters by applying our recently developed Bayesian Information Criterion-Super K means (BIC-SKmeans) algorithm^15^ (Supplementary Fig. 1g, h). As shown by the marker genes’ RNA-seq expression, the four whole-transcriptome sample clusters correspond to the inner cell mass, pluripotent epiblast, definitive ectoderm (late epiblast cells), neuroectoderm, and neural progenitor stages (Fig. 1b and Supplementary Data 1). Such cpRNA-seq data was then used to guide our selection of time points for scRNA-seq to examine intercellular heterogeneity during the differentiation process.

### Saturation of sequencing depth to detect heterogeneity

To examine whether there is any heterogeneity during neural commitment at the single-cell level, we performed scRNA-seq analysis as using a well-established protocol^16^ for 64 single cells at eight time points (eight single cells for each, two timepoints per stage identified by the above cpRNA-seq profiles) during neural differentiation from mESCs, and also performed cpRNA-seq at the same time points in parallel (Fig. 1a). A minimum of 20 million reads were generated using an Illumina HiSeq2000 sequencer for each library to reach a saturation level (Supplementary Fig. 2a).

Comparing the similarity of scRNA-seq sample average profiles to their matching cpRNA-seq samples at the same time point or stage offers an opportunity to access how many samples are needed to capture intercellular heterogeneity at a time point or within a stage. That is, if adding more scRNA-seq samples no longer increases scRNA-seq average to cpRNA-seq correlation, sample contribution to intercellular heterogeneity has been saturated. For the mESC neural differentiation, 16 samples per stage already reached such saturation level, while 8 samples per stage closely approached saturation (Supplementary Fig. 2b for stage and Supplementary Fig. 2c for time points). This function is included in our iCpSc package as “iCpSc.samplingSaturation.”

To further validate the effectiveness of our saturation analysis method, we tested it on simulated data. As demonstrated in Fig. 1c, on simulated cpRNA-seq data generated using a linear mixture model from 6 of our scRNA-seq cells with 1:2:3 ratio, 15 cells with 1:2:3:4:5 ratio, or 34 cells with 3:4:5:6:7:9 ratio, in all cases, the sampling reaches perfect saturation when all cells are sampled, and approaches a close to saturation point with small variations at 4, 12, and 21 cells, respectively, demonstrating a theoretical robustness of the iCpSc.saturation analysis method. Similar saturation points can be identified using two public datasets that contain both time-series single-cell RNA-seq and matching bulk RNA-seq data—human skeletal muscle myoblast differentiation (GSE52529) and human neural differentiation (GSE86894), suggesting that our package can be generalized to other data, and is not dataset specific (Fig. 1c).

Deep sequencing depth per cell can minimize or eliminate the cell–cell variation introduced technically due to the sampling effect. So far this sampling effect has not been specifically examined. Our iCpSc method provides an opportunity to unbiasedly investigate such an issue using cpRNA-seq as the reference. By sampling three datasets to different coverage levels, we show that sequence depth indeed has a large impact on the number of samples required to reach a saturation level to match the cell population profiles, the lower depth requires a much larger number of cells to sequenced (Fig. 1d).

Analyses using simulated data show a similar trend (Supplementary Fig. 3), although rare cell types take deeper than usual sequencing depth to uncover (Supplementary Fig. 3), and are more susceptible to unbalance cell population expansion (Supplementary Fig. 4). In these analyses, we first simulated four subtypes of cells at various compositions with the rarest subtype comprising 5% of total 20,000 or 50,000 cells^17^ and randomly sampled them at different sampling depths (Supplementary Fig. 3 and Methods section). All four subtypes could be seen from 0.05% sampling depth in both simulated datasets (10 cells in 20,000 cells or 25 cells in the 50,000 cells; for a less rare 10% population, 0.02% or 10 cells in 50,000 cells), and the compositions of the four cell types become the same as the actual composition starting from the sampling depth of 0.5% (Supplementary Fig. 3c). We also compared the slope of similarity to bulk reference transcriptome (using Pearson's correlation coefficient (PCC)) curve for randomly sampled cells, with that for cells sampled with the exact same composition as in the initial population (Supplementary Fig. 3d). A discrepancy between the two disappears at ~0.1–0.2% of sampling depth, which also indicates the minimal depth needed to capture the original composition of the cells.

To see if compositional changes over time could lead to the inference of incorrect time points when mapping to a bulk reference sample, we simulated two equally sized sub-populations that are distinct on the transcriptome level, i.e., 20% of the genes differ significantly (*p* < 0.05, generated using the splatter package^17^). Then the two sub-populations of single cells change at each timepoint, a total of five timepoints with 1% of genes change significantly at each timepoint (*p* < 0.05, generated using the splatter package). In addition, one of the populations strongly expands, while the other one remains constant, such that at the final time point the composition is 95% vs. 5%. Using the iCpSc.CpToScTime package to infer the temporal order/trajectory of single cells, we found in the no-expansion model that the inferred trajectories correlate highly with the actual orders for both minor and major cell types. In the expansion model, for the minor cell type, the correlations (Spearman's rank correlation coefficient (RCC)) decrease significantly, while there is little decrease for the major cell type during expansion (Supplementary Fig. 4).

### Heterogeneity of neural commitment revealed by scRNA-seq

We found substantial differences in gene expression between individual cells at the same time point (PCC range: 0.35–0.71; Fig. 1e and Supplementary Fig. 5), which reflected the extensive cell–cell variation at the transcriptome level. In contrast, the average gene expression levels of the eight single-cell groups at each of the eight timepoints are well correlated with the gene expression levels measured by cpRNA-seq (PCC range: 0.71–0.79; Fig. 1e), particularly, at the initial and late stages of mESC neural differentiation (Fig. 1f), suggesting higher conformity of single cells at these end points. Unsupervised hierarchical clustering (Fig. 1g) or principal component analysis (Fig. 1h) revealed similar patterns. In addition, we observed a clear transition trajectory from mESCs to neural progenitors, as well as differential rates of commitment across individual cells from the same time point (Fig. 1h), where single cells are ordered similarly as shown by global hierarchical clustering (Fig. 1f).

### A differentiation timer in single cells

Intriguingly, according to our single-cell data—except for a single outlier cell that remained stuck in the original stage at the very last time point (the “S4b-16” cell)—all cells seemed to only differ in temporal position along the differentiation trajectory (Figs. 1f, h). That is, no other new state appears which branches away from the four major stages (S1–S4, Fig. 1f). This suggests that the intercellular heterogeneity largely reflects the speed of differentiation, rather than a lockdown at certain stages without proper progression.

We sought to perform analysis of single cells according to their progression. There are many methods developed to infer pseudotime trajectories of single cells based on scRNA-seq. However, the pseudotime is only an order of cells, rather than a reflection of real differentiation time, and such pseudotime analyses are limited by confounding factors, particularly the cell cycle^3, 14^. Unfortunately, we cannot be sure which factors cause the misalignment of inter-cell distance-based trajectory reconstruction. The cell cycle often appears as a confounding factor and its removal by the scLVM package is the currently available resolution for analysis. However, as the cell cycle is emerging as a key regulatory component of differentiation timing^5–7^, removing cell-cycle-related gene expression is not a viable approach. It was therefore necessary to develop a new method to analyze scRNA-seq differentiation data.

**Fig. 2:**
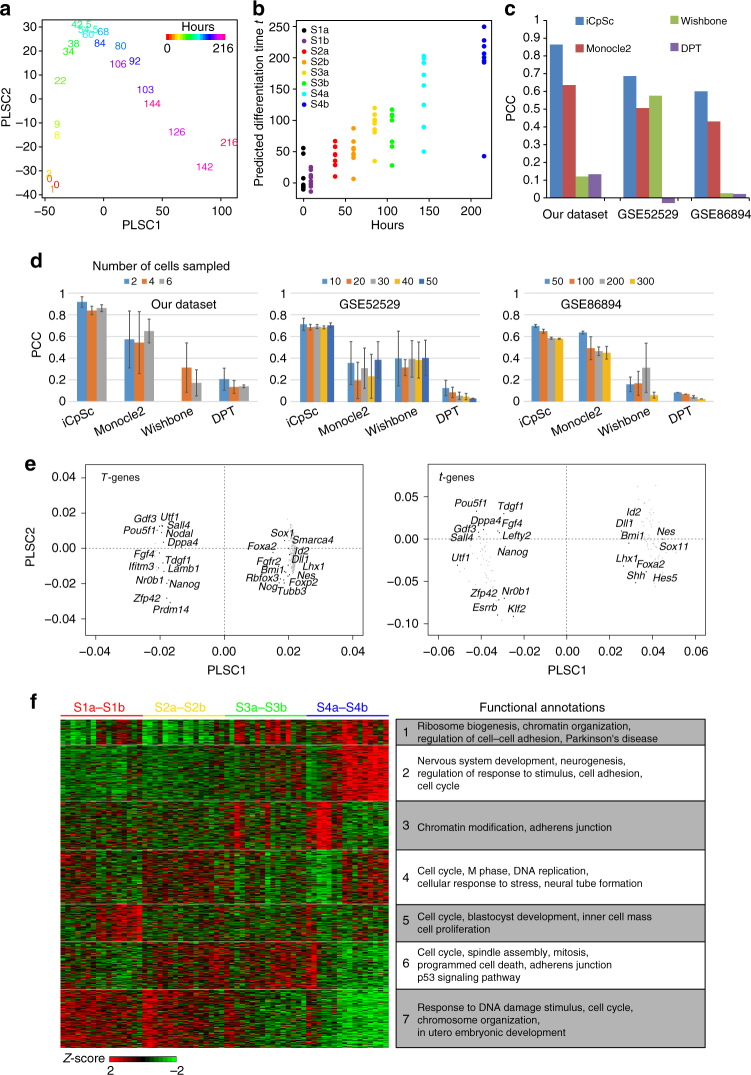
Neural differentiation timing in single cells. **a** Temporal order displayed by cell population samples when projected on the first and second partial least-square (PLS) regression components of cell population RNA-seq profiles. **b** Single cells ordered by the differentiation time *t* predicted by the PLSC1 and 2 derived linear model (see Methods). Cells with the same color were obtained at the same experimental time point. **c** Similarity between real sample collection time and the predicted time and given by four different (pseudo-)time prediction methods on the mESC neural differentiation dataset or two public datasets. **d** Robustness of four (pseudo-)time prediction methods. Similarity between real sample collection time and the predicted time and given by four different (pseudo-)time prediction methods on the mESC neural differentiation dataset or two public datasets, in randomly selected sample sets with different numbers of cells sampled for each time point. Error bars indicate the standard deviation of five sampling tests. **e** The projections onto PLSC1 and PLSC2 for selected genes: genes (gray dots) that have the top 10 strongest expression correlations with the fitted differentiation time *T* in cell population (left) or the predicted differentiation time *t* in single cells (right), and development-related marker genes that have |PCC| > 0.6 with *T* or *t* are shown as black dots with gene names. **f** The expression pattern of predicted differentiation time-related genes, the “*t1–4*-genes”, clustered by BIC-Skmeans and their functional annotations

We developed the “iCpSc.CpToScTime” utility which is based on first extracting a reference differentiation time from cpRNA-seq data and applying it to scRNA-seq data. To do this, we used a modeling method similar to that in our recent image-based biological age prediction^18^. We first extracted partial least-square principle components (PLSC) that show a linear relationship to the actual differentiation time using the cpRNA-seq data. For our mESC neural differentiation data the first two components, PLSC1 and PLSC2, together explained 73% of the variance associated with time. These components were well separated and ordered cell population samples according to their time points (Fig. 2a). We define the PLSC1 and PLSC2 linear model fitted differentiation time as *T*, which is highly correlated to actual time (PCC = 0.979). Next, based on PLSC1 and PLSC2, we generated a linear model to predict the differentiation time (denoted as *t*) of each single cell by fitting it to the single-cell gene expressions’ projections (loadings) on PLSC1 and PLSC2 (Fig. 2b, Supplementary Fig. 6a, and Methods section). The ordering of the single cells by their predicted differentiation time, although derived from an external reference, was in general similar to the order given by Monocle2 (Spearman’s RCC = 0.92; Supplementary Fig. 6b, c). We further compared our methods with the three existing methods, diffusion pseudotime^19^, Wishbone^20^, and Monocle2^1, 21^, using the similarity to sample collection time as an evaluation parameter. iCpSc predicted times show the highest correlations to the real collection time as compared to the other methods (Fig. 2c). Similar results were observed for the published human skeletal muscle myoblast differentiation and neural differentiation dataset (Fig. 2c). It should be noted that other than iCpSc, the three other methods can only predict pseudotime, instead of real time. Moreover, we examined the robustness of performance by each of the four methods through sampling cells. For all three datasets, iCpSc shows not only highest correlation between predicted time and real collection time, but also the most robust pattern among different sampled sets of cells (Fig. 2d).

Plotting predicted time against scPLS2, which captures the largest variances that is not the strongest correlate with predicted time, also allows iCpSc to detect branching trajectories (Supplementary Fig. 6d).

To identify the genes contributing to the differentiation timing, we defined the 2721 genes that are significantly correlated to *T* as “*T*-genes” in cpRNA-seq data (*p*-value < 0.003; Supplementary Fig. 7a, Supplementary Data 2, and Methods section). Meanwhile, we defined that the 1035 genes significantly correlated to predicted differentiation time *t* of a single cell (*p*-value < 0.001; Supplementary Fig. 7a and Supplementary Data 2) in scRNA-seq data as “*t*-genes” (Fig. 2e). There were 562 genes that overlapped between the *T-* and *t-*genes. In addition, correlation in the opposite direction to the time *T* or *t*-axis revealed pluripotency genes including *Pou5f1*, *Utf1*, and *Sall4*, and the neural commitment markers such as *Nestin*, *Sox11*, and *Hes5* (Fig. 2e).

Then, by identifying genes whose expression level in single cells are linearly related to the predicted differentiation time at each differentiation stage, we tried to uncover a gene expression signature of differentiation timing within each stage. We refer to these genes as “*t1*-genes”, “*t2*-genes”, “*t3*-genes,” and “*t4*-genes”, respectively, and “*t1–4*,*”* collectively (Supplementary Fig. 7b, Supplementary Data 2, and Methods). Surprisingly, *t*- and *t1–4*-genes are highly enriched for cell-cycle-related genes, whereas *T*- and *T1–4*-genes do not enrich cell-cycle-related genes (Fig. 2f, Supplementary Fig. 7b–e, and Supplementary Data 3). Cell-cycle stage is known to play an important role in regulating cell differentiation, e.g., a longer G1 phase promotes differentiation^5–7^. However, it is not clear why and how the cell cycle is involved. Whether the speed of neural commitment and differentiation is actively timed, and further, how the speed and timing for any differentiation process is measured and controlled remains a mystery. It is therefore intriguing that, as suggested by our results, the cell cycle might be a timer, or at least a marker of a timer, for the differentiation process.

### Inferring regulatory networks modulating heterogeneity

**Fig. 3:**
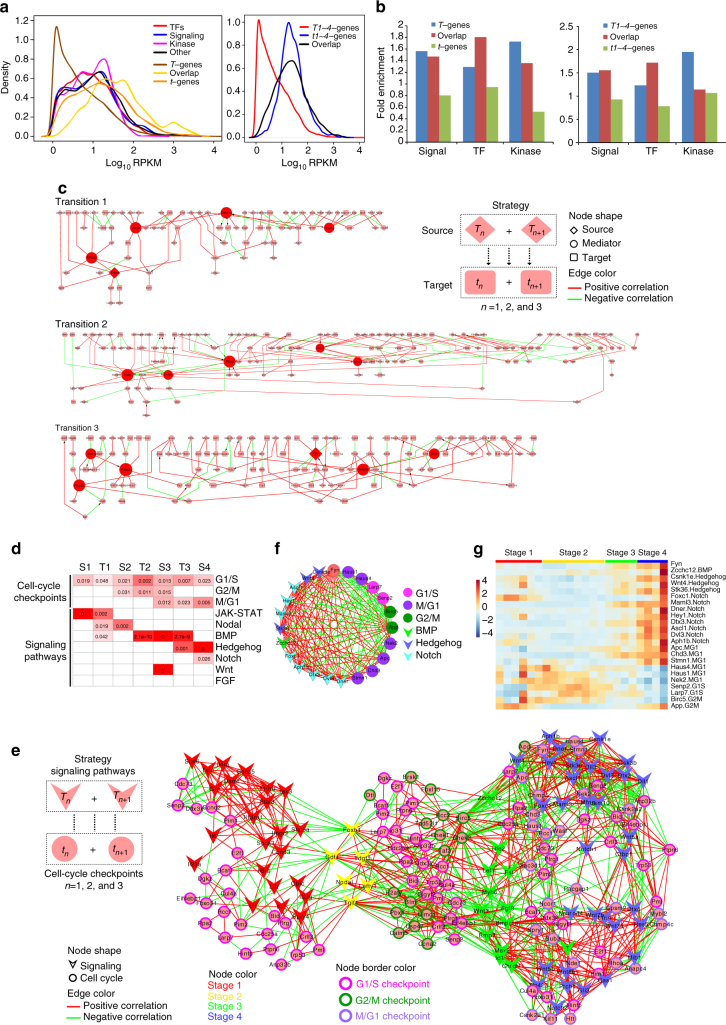
Inferring regulatory events for mESC neural differentiation timing. **a** Expression level distribution of signaling genes, kinases, and transcription factors, compared with scRNA-seq detectable genes and undetectable cpDEGs (left panel), *T-/T1–4*-genes, *t-/t1–4*-genes, or their overlapping genes (right panel) in the cell population RNA-seq data. **b** Fold enrichment for signaling genes, kinases, and transcription factors in scRNA-seq detectable and undetectable cpDEGs, in *T-/T1–4*-genes, *t-/t1–4-*genes, and their overlapping genes. **c** The largest component of each stage transition eResponseNet. The graphic legend shows the eResponseNet input data. Nodes with degree > 4 (top 5%) are labeled as hubs (large nodes). Red/green edge color represents positive/negative correlation (|PCC| > 0.6) between the two nodes’ expression profiles in the cell population RNA-seq data. See graphic legend for node annotations. **d** Significance of enrichment for three cell-cycle checkpoints and seven development-related signaling pathways’ targets in four stages and three stage transitions. Significances of enrichment for pathways members are shown in Supplementary Fig. 8c. Significant enrichment is shown by red blocks, with white representing insignificant. Fisher’s exact test was used to test cell-cycle checkpoint enrichment in *t*_*n*_-genes (*n* = 1, 2, 3, and 4) and in genes of each transition eResponseNet (Bonferroni-corrected *p*-value < 0.05). GSEA against the rank list sorted by expression levels of all expressed genes, and Fisher’s exact test are used to test signaling pathway enrichment in each stage and in each transition eResponseNet over the whole genome, respectively (GSEA FDR < 0.05, Bonferroni-corrected *p*-value < 0.05, see Methods section). For column names, “S” stands for “Stage” and “T” for “Transition”. **e** The CSI network among the *T*_*n*_- or *t*_*n*_-genes belonging to enriched signaling pathways and cell-cycle checkpoints, respectively (*n* = 1, 2, 3, and 4). Gene expression PCC-derived CSIs are calculated based on cell population RNA-seq expression values. The stage of a gene is defined by the stage where its pathway is activated (see Methods section). **f** Subnetwork of Fyn from **e**. Node shapes indicate cell-cycle checkpoints or signaling pathways. Node colors represent different gene categories. **g** Expression patterns of genes in **f** network during mESC neural differentiation

One purpose of scRNA-seq is to infer regulatory networks that account for the intercellular heterogeneity. However, this is often hindered by the low amount of RNA detectable by scRNA-seq and that signaling genes are often expressed at low levels, hence not detectable by scRNA-seq. Even when detected, because of their low levels, analyses are more affected by technical noise^3^. We noticed that about 30% of differentially expressed genes in matching cpRNA-seq were not detected by scRNA-seq. Interestingly, the *T*-genes/*T1–4*-genes that were not detected in single cells had significantly lower expression levels compared with *T*-genes/*T1–4*-genes detectable in single cells (Fig. 3a, right panel) and that the undetectable *T*-genes/*T1–4*-genes were much more enriched for transcription factors, kinases, and signaling genes over the genome background (Fig. 3b). This observation hints at a possibility that the *T*-genes/*T1–4*-genes that are not expressed in single cells might act as upstream signals to regulate the differentiation time-related genes at the single-cell level. To identify potential pathways linking the signaling genes in cpRNA-seq data to the intercellular heterogeneity marker genes in the scRNA-seq data, we adapted the eResponseNet package^22^ to accept cpRNA-seq genes as source nodes and scRNA-seq genes as target nodes, and included the method in the iCpSc package. The eResponseNet algorithm searches for a subnetwork that carries the largest information flow between the source and target gene sets based on an interaction network template and the flow weight of the edges^22^, and thus can potentially be adapted to integrate potential regulators of cpRNA-seq to differentially expressed scRNA-seq genes.

For each mESC neural differentiation stage transition, we used the *t*_*n*_ and *t*_*n+1*_ genes as molecular signatures or target nodes (*n* = 1, 2, and 3), and *T*_*n*_ *and T*_*n*+1_ genes detectable (expressed) in cell populations but undetectable (unexpressed) in the single cells as source nodes (Fig. 3c and Supplementary Fig. 8a). Here we defined the flow weight of edges as the PCC between two genes’ expression profiles in the cpRNA-seq data in the two consecutive stages *n* and *n *+ 1 for each stage transition (see Methods section).

We noticed that cell-cycle-related genes were much more enriched (3.1-fold enriched) in the eResponseNet network than in the *t1–4*-genes (1.8-fold enriched) even though in both they were statistically significantly higher than the genome background (Supplementary Fig. 8b, Fisher’s exact test *p*-value = 6.5e−54 and *p*-value = 1.7e−13, respectively), which further suggests potentially critical roles of the cell cycle during neural differentiation.

To further identify the roles of the cell cycle in differentiation control, we collected G1/S, G2/M, and M/G1 (spindle and metaphase-to-anaphase) checkpoint-related genes from GO to test their enrichment in *t1–4*-genes for each stage and in the eResponseNet for each stage transition. We found that G1/S checkpoint genes are enriched in all stages and transitions, while G2/M checkpoint genes are only enriched in the second transition and its neighboring stages (Stages 2 and 3), and M/G1 checkpoint genes are only enriched in the third transition and its neighboring stages (Stages 3 and 4, Bonferroni-corrected *p*-value < 0.05 by Fisher’s exact test; Fig. 3d). To infer the interplay between cell-cycle checkpoints and different signaling pathways in controlling each transition, we calculated the enrichment for seven development-related pathways in each stage or transition eResponseNet network. The activated signaling pathways in each stage or transition eResponseNet were determined based on the enrichment of both the signaling pathways’ members and upregulated targets (Supplementary Fig. 8c and Methods section). The enrichment analysis indicates that JAK-STAT, Noda,l and BMP pathways are activated in Stages 1, 2, and 3, respectively, and Notch and Hedgehog pathways are both activated in Stage 4 (false discovery rate (FDR) < 0.25 by Gene Set Enrichment Analysis (GSEA), Fig. 3d, Supplementary Fig. 8c, and Methods). Consistent with the identified eResponseNet results representing potential regulatory networks, they were much more enriched in signaling genes than either the source or target gene sets (Supplementary Table 2). Furthermore, we found that the interactions between 13 of the possible 21 pairs of signaling pathway (*x* genes) and checkpoint (*y* genes) are significantly more than randomly selected *x* × *y* size gene sets (1000 permutations for each test, Supplementary Table 3).

To confirm links between these signaling pathways and cell-cycle events without any network template or prior knowledge, we generated a co-expression network using the Connection Specificity Index (CSI)^23^ among the genes in these pathways and checkpoints. Interestingly, and consistent with the timing-associated cell-cycle phases we observed above (Fig. 3d), the signaling pathways active in the second transition are highly connected to G1/S and G2/M cell-cycle checkpoint genes, whereas active signaling pathways of the last transition are highly connected to both the G1/S checkpoint and M/G1 checkpoint genes (Fig. 3e and Supplementary Table 3). For example, the G1/S cyclin, cyclin E1 (*Ccne1*) is correlated with the Nodal pathway gene *Tdgf1* and negatively correlated with BMP receptor *Bmpr1a* in the second transition, while, in the third transition, *Ccne1* co-expressed with the BMP signaling molecule *Fgf8* and was anti-correlated with many Notch signaling genes, such as *Notch1*, *Hey1*, and *Dtx3*. To further test the eResponseNet and CSI networks, we focused on the hubs that are connected to cell-cycle genes to see whether they exert their effects on timing and neural lineage commitment through regulating the cell cycle (Figs. 3c, e and Supplementary Fig. 8b, Supplementary Table 4, and Methods). To this end, we observed that cell-cycle genes are 1.6- and 2.5-fold more enriched among interactors of the hub genes than those of non-hub genes, or 3.7- and 19.8-fold than genome average, in the eResponseNet and CSI networks, respectively. Interestingly, *Fyn* is one such hub in both the eResponseNet and CSI networks, connecting to mitotic checkpoint genes and Notch pathway genes (Fig. 3f). Since the Notch pathway is a key controller for neural precursor cell (NPC) identity, which is the major cell fate after the third transition, this observation hints at a regulatory role of *Fyn* in the final transition to NPC fate.

### Experimental validation of differentiation timing regulation by Fyn

**Fig. 4:**
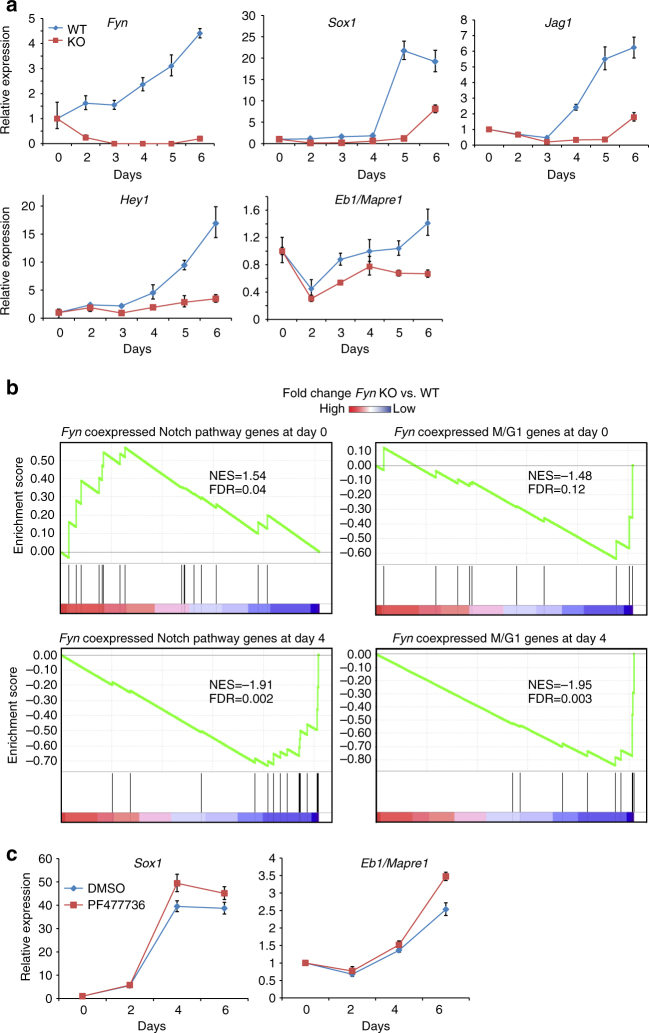
Regulation of neural differentiation timing and the cell cycle by Fyn. **a** Expression levels of *Fyn*, differentiation markers *Sox1, Jag1*, and *Hey1* and M-phase markers *Eb1*/*Mapre1* in wild-type (WT) and *Fyn* KO E14 cells from days 0 to 6 of neural differentiation. Error bars indicate standard deviation of three technical replicates. **b** RNA-seq of WT and *Fyn* KO E14 cells revealed a significant downregulation of Fyn-co-expressed Notch pathway genes and M/G1-phase genes (genes in Fig. 3f, g) on day 4 of mESC neural differentiation. **c** Expression levels of *Sox1* and *Eb1/Mapre1*, with or without G2/M arrest inhibitor PF477736 treatment from days 2 to 4 of neural differentiation. Error bars indicate standard deviations of three technical replicates

We noticed that G1/S and G2/M checkpoints genes are significantly enriched at transition 2, and M/G1 genes are enriched at transition 3 in the eResponseNet and CSI networks. Meanwhile, *Fyn* is connected to multiple G1/S, G2/M, and M/G1 checkpoint genes, in addition to Hedgehog and Notch pathway genes in the CSI network (Fig. 3e, f). It is also a mediator node at transition 2 and a hub mediator at transition 3 in the eResponseNet network (Fig. 3c). Additionally, induction of *Fyn* occurs immediately before the appearance of NPCs at transition 2, and its expression increases in parallel with Notch, Hedgehog, and many M/G1 and G2/M genes (Fig. 3g). These led us to ask whether Fyn can act on cell-cycle checkpoints and control the speed/timing of mESC differentiation to NPCs. To do this, we generated a *Fyn* KO in mESCs with CRISPR/Cas9 (Fig. 4a and Methods). As predicted, we found that *Fyn* KO delayed *Sox1* induction, and hence neural differentiation. Given the strong correlation between *Fyn* and Notch signaling genes in the CSI network (Fig. 3f), we examined whether Fyn may regulate the Notch pathway. *Fyn* KO not only delayed the induction the *Jag1* gene, which encodes the canonical Notch1 ligand Jagged1, but also the transcription factor *Hey1*, the canonical target of the Notch pathway (Fig. 4a and Supplementary Fig. 9a).

Pilaz et al.^24^ have found that the prolonged mitosis of NPCs promotes neuron generation in the developing mouse brain. Meanwhile, *Fyn* overexpression has been known to accelerate progression from prometaphase to M phase and increase M-phase cell numbers^25^. Consistently, we found that *Fyn* KO significantly decreased expression of the M-phase marker gene *Eb1/Mapre1* ^25^ (Fig. 4a and Supplementary Fig. 9a). We further confirmed an overall downregulation of the Fyn-co-expressed Notch pathway and M-phase gene expression upon Fyn KO by RNA-seq (Fig. 4b).

Consistent with the finding that the M-phase elongation can accelerate the neural differentiation process and that *Fyn* KO undermines M phase and neural differentiation, treating the cells with a drug, PF477736, that can release G2 arrest and elongate M phase by inhibiting CHK1 and CHK2^26^ had the opposite effect on cell-cycle stage and differentiation marker gene expression as *Fyn* KO (Fig. 4c and Supplementary Fig. 9b). Together, these results validate our iCpSc prediction of *Fyn* as a novel regulator of the cell cycle, the timing and speed of neural commitment, and Notch pathway activity, and that the cell cycle, particularly M phase, is involved in the timing/speed of neural differentiation.

## Discussion

Single-cell RNA-seq has become a standard method to identify intercellular heterogeneity during development and reprogramming. It has many advantages that are unmatched by cpRNA-seq. Primarily, by averaging the expression profiles of individual cells, cpRNA-seq masks intercellular transcriptome heterogeneity. We found that averaged transcriptomes failed to reveal cell-cycle genes as the prime correlates to the single cells’ differentiation time, which were identified only in the single cell transcriptomes.

However, scRNA-seq analysis methods are far from complete or perfect^3^. To start with, even the minimal number of scRNA-seq samples needed to reliably detect intercellular heterogeneity has not been examined. Here we used matched time point cell population data to provide an external standard that addresses this question. Our “iCpSc.samplingSaturation” utility compares the scRNA-seq average to the cpRNA-seq data at the same time point, and plots a saturation curve, to determine whether the sampling is sufficiently saturated to robustly capture major intercellular heterogeneity. It should be noted that our cpRNA-seq- based saturation analysis is meant to assess the MINIMAL number of cells to be sequenced in order to reliably capture intercellular heterogeneity in a biological process, such as the differentiation or dedifferentiation process. For other purposes, e.g., to identify rare cell populations, obviously the more cells sequenced, the easier it is to capture the rare cell populations, which are made possible by the ever decreasing cost and increasing depth of new scRNA-seq technologies. According to our sampling results on simulated data, this minimal number of cells might be an optimistic estimate for rare cell populations (< 5%), especially when cell population expansions are unbalanced (Supplementary Figs. 3, 4).

Existing single-cell analysis methods, including the popular single-cell trajectory reconstruction methods, suffer from many confounding factors, such as the cell-cycle and other hidden variables. Consequently, cells might be aligned to a trajectory based on confounding factors, rather than true differentiation or developmental time. One solution is to remove such confounding factors one by one. However, not all confounding factors are known beforehand (missed false negative factors that give false positive association), and not all assumed confounding factors are actually irrelevant to the process under study, e.g., differentiation and development (factors that give false negative association if removed). In particular, the cell-cycle regulation has recently been shown to be an important causal and regulatory event to many differentiation and development processes^5–7^. Simply removing it will cause true regulatory events to be missed. Our approach (coded by the “iCpSc.CpToScTime” utility) using matched cell population transcriptomes of the same process to build a reference differentiation time model, and applying the reference to single cell transcriptomes, solves this dilemma in an unbiased way and allows reliable identification of cell-cycle events associated with differentiation timing. Furthermore, the reference time model-derived prediction actually gives the scale of differentiation time, unlike the pseudotime given by current single-cell-only based trajectory analysis methods, which are orderings, but not scales of time.

In addition, cpRNA-seq- and scRNA-seq-coupled network analysis further allows identification of regulators giving rise to cell heterogeneity, instead of providing only a measure or markers for heterogeneity. Using the “iCpSc.eResponseNet” utility, we inferred cell-cycle checkpoint genes that potentially regulate mESC neural differentiation timing, and experimentally validated an iCpSc predicted role of the network hub *Fyn* to regulate cell cycle and Notch signaling, and mESC differentiation to NPCs.

We expect future single-cell analyses in other biological systems will further discover and characterize the cell cycle and cell-cycle checkpoints as key factors in the timing of various state transitions. In this respect our method of integrating analysis of temporally matched cpRNA-seq and scRNA-seq data with our comprehensive computational analysis package, iCpSc, provides an effective and unbiased approach for assessing sampling depth, identifying developmental trajectory and timing related regulatory events, though it is not limited to only this application.

## Methods

### Cell culture

Mouse R1 ES cells, originally from Janet Rossant’s laboratory, were propagated using a standard method^27^ and then induced into neural progenitors using monolayer differentiation and SFEB (serum-free floating culture of embryoid body-like aggregates) differentiation protocols, respectively^28, 29^. ES cells were passaged once under feeder-free conditions to remove feeders. For monolayer differentiation, ES cells were dissociated with TrypLE (Invitrogen) and resuspended in N2B27 medium. After that, ES cells were plated onto gelatin-coated dishes at a density of 5 × 10^4^/ml and the medium was changed every other day. On day 6, cells were replated onto poly-d-lysine (PDL)/laminin/fibronectin-coated dishes at a density of 0.5–1.5 × 10^4^/cm^2^ ^29^. For SFEB differentiation, ES cells were dissociated and resuspended in GKSR medium at a density of 5 × 10^4^/ml. Then the cells were seeded into Petri dishes (10 ml). On day 6, cells were dissociated and resuspended in the GMEM-N2 medium. Then the cells were replated onto PDL/laminin/fibronectin coated dishes at a density of 0.5–1.5 × 10^4^/cm^2^ ^28^). The small molecule inhibitors were added from day 2 of monolayer differentiation. These include 0.5 μM or 1 μM PP1, and 0.1 μM PF477736. The vehicle dimethyl sulfoxide was used as control.

### Flow cytometry

Cells (~1 × 10^6^) were suspended in cell staining buffer and incubated with anti-O4-APC antibody (R&D) for 30 min. Then the cells were washed twice by staining buffer, fixed by fixation buffer, resuspended in permeabilization/wash buffer (R&D), and finally stained with anti-GFAP-PE (BD) and anti-TUBB3-AF488 (Biolegend) antibodies. Data were collected from one million to three million cells with FACSCalibur (BD) and were analyzed with the FlowJo software.

### Real-time PCR assays

RNA was isolated using the RNAeasy mini kit (Qiagen) and reverse transcription was performed using SuperScript III (Invitrogen). Real-time PCR was performed on Mx3000P detection system (Stratagene) using TransStart Green qPCR SuperMix (Transgen). Values were normalized against *Gapdh*.

### High-throughput real-time PCR

The experiment was performed using the 48.48 dynamic array (Fluidigm Corporation, CA, USA) according to the manufacturer’s protocol (PN 100-1208 A4). Briefly, cDNA samples were diluted 1:10 with ddH_2_O. Individual primer sets were pooled to a final concentration of 200 nM for each assay. Diluted cDNAs were combined with mixed primers and TaqMan PreAmp Master Mix (Applied Biosystems) for 14 cycles of amplification. Unincorporated primers were removed by Exonuclease I. Products were diluted to 10–20-fold. Then the sample Pre-Mix solutions and Assay solutions were prepared and loaded into chip using IFC Controller MX. Data collection and data analysis were done using the Fluidigm BioMark System. Primers are listed in Supplementary Table 1.

### Single-cell cDNA preparation and RNA sequencing

Single-cell cDNA was prepared using a published protocol^16^. Quality control of double-stranded cDNA was carried out on an Agilent 2100 chip (Agilent Technologies) before library construction. RNA-seq libraries were then prepared according to the protocol of Nextera XT DNA Sample Prep Kit (Illumina). Individual cells were manually picked and transferred into cell lysis buffer. Reverse transcription was performed using the whole cell lysate. After poly(A) was added to the 3′ end of first-strand cDNAs, second-strand cDNA was synthesized. Then the single-cell cDNAs were amplified by PCR for 20 + 9 cycles.

### RNA sequencing

Quality control of double-strand cDNAs was carried out with an Agilent 2100 system (Agilent Technologies) before library construction. RNA-seq libraries were then prepared according to the protocol of Nextera XT DNA Sample Prep Kit (Illumina). Briefly, 100ng cDNAs were fragmented and ligated with adapters. Twelve cycles of PCR were performed to amplify the fragments and add indexes. Primers and very short library fragments were removed by AMPure XP beads (Beckman Coulter). After that, size selection was done by agarose gel purification and the purified samples were quantified by qPCR and Qubit. Normalized libraries were pooled and sequenced on Illumina HiSeq2000.

### Cell population and single-cell RNA-seq data preprocessing

We obtained 100 bp single-end reads by HiSeq2000, then mapped them to the mouse genome build mm9 using Tophat v.1.4.1 with the following parameters: -p 20 -g 1 -N 6 -- no-novel-juncs -G^30^. We calculated RPKM as expression level using Cufflinks v.1.3.0 with default parameters^31^. For single-cell RNA-seq data, we first used the quantile normalization function in R to normalize gene expression levels in different cells. Next, we discarded genes that do not have RPKM > 0.5 in at least eight individual cells within in all 64 single cells, then transformed expression levels by log_10_(RPKM + 1). The saturation curves for single cells are shown in Supplementary Fig. 2A.

### Identification of differentially expressed genes

For cell population data, we used Cuffdiff to identify differentially expressed genes between every two samples (*p*-value < 0.01)^32^.

### Clustering analysis

*Z*-score normalized RPKM were used for BIC-SKmeans clustering^15^, where the optimal numbers of clusters were determined by adjusting lambda.

### Functional enrichment analysis

Functional enrichment (GO annotation, KEGG, and Wiki Pathway) of gene sets with different expression patterns was performed using DAVID v.6.7 and findGO.pl program in Homer^33–35^.

### Testing scRNA-seq sampling saturation

To test the saturation of scRNA-seq for capturing intercellular heterogeneity, we first randomly selected 1 to *n* single cells (*n* is the total number of single cells for each time point or stage), calculated the average expression level of each gene among selected single cells, removed lowly expressed genes by requiring RPKM > 0.5 in at least eight samples, and then calculated the PCC between the average scRNA-seq gene expression profile and the cpRNA-seq profile for each time point or stage. To avoid an unnecessary exhaustive search when n is large, we set an upper limit (30 by default) for randomly selecting combinations in our utility.

### Generation of simulated cpRNA-seq data

To generate a simulated cpRNA-seq data, RPKMs for each gene from scRNA-seq data are summed at a given ratio of different cells, e.g., 1:2:3, representing six cells’ RPKMs of three single cells.

### Sampling cpRNA-seq data to simulate scRNA-seq data

To test the effect of limited sampling, scRNA-seq reads are sampled at designated depth, e.g., 0.1 M for 0.1 million reads sampled and then mapped to the genome, and RPKM were calculated for all genes. Genes with RPKM > 0.5 in 30% of the scRNA-seq samples were used to calculate PCC of the total RPKMs of all sampled RNA-seq data to the corresponding cpRNA-seq data.

### PLS analysis

To detect time-associated components, PLS analysis was applied to the differentially expressed genes from the cpRNA-seq data (cpDEGs) and regressed to the sample collection time. PLS maximizes the covariance between the samples’ component values and the sample collection time. R packages were used for PLS^36^.

### Alignment of single-cell temporal orders

We use a model similar to our three-dimensional image-based biological age predictor^18^. After confirming that PLSC1 and PLSC2 in the cell population data linearly predict time, we ordered single cells based on the PLS model of cell population. First, we built a PLS model from the cell population data by using only the 1593 genes that overlap between cell population DEGs and single-cell expressed genes (RPKM > 0.5 in at least eight samples the RNA-seq data and have non-zero value for more than half single cells). We first extract the first and second PLS components most correlated with the actual sample collection time using the “pls” package in R, where *Wn* in formula ${\mathrm{PLSC}}n = X \cdot Wn$ is solved for the variables matrix *X*, the cell population expression value matrix for the above-described 1593 genes, and build a linear model by $T = q1*{\mathrm{PLSC1}} + q2*{\mathrm{PLSC2}} + e$, by regressing PLSC1 and PLSC2 against the sampling time in cell population. The above functions were packaged into the “iCpSc.CpToScTime” utility.

We define genes whose expression profiles across cell population time point samples have |PCC| > 0.6 (corresponding to *p*-value < 0.003 based on sample randomization) to *T* in the above linear model as “*T*-genes” in cpRNA-seq data. Then we use the model to predict the differentiation time of a single cell by replacing *X* in in formula ${\mathrm{PLSC}}n = X \cdot Wn$ by the single-cell expression values of the same 1593 genes as the variables matrix *X*. Next, to correct the batch effect between cell population and single-cell data, we transformed the predicted time by the linear model *t = aT + b*, where *a* and *b* are determined by regressing *T* against the actual sampling time of each single cell. This linearly adjusted predicted differentiation time was then used to temporally order all single cells. We define genes whose expression profiles across single cells have |PCC| > 0.4 (corresponding to *p*-value < 0.001 based on sample randomization) to model predicted time *t* as “*t*-genes” in single-cell RNA-seq data, and within each stage, “*t1*-genes”, “*t2*-genes”, “*t3*-genes,” and “*t4*-genes” across 16 single cells, respectively (*p*-value < 0.05 based on sample randomization for each set).

### Cell-cycle checkpoint genes and enrichment analysis

We collected G1/S, G2/M, and M/G1 (spindle and metaphase-to-anaphase) checkpoint-related genes from GO. We then used Fisher’s exact test to examine their enrichment significance in each stage-specific *t*_*n*_-genes (*n* = 1, 2, 3, and 4) and in each transition eResponseNets (Bonferroni- corrected *p*-value < 0.05).

### Enrichment test of cell-cycle checkpoint genes

We collected G1/S, G2/M and M/G1 (spindle and metaphase-to-anaphase) checkpoint genes from Gene Ontology (GO), and tested their enrichment against the rank of fold changes of all genes upon PP2 treatment in primary mammary tumor cells (GSE50517) and Notch1 induction in mESCs (GSE15268), respectively, by GSEA.

### eResponseNet network analysis

The eResponseNet package^22^ was used to identify the regulators of the predicted differentiation time related gene expression changes at the single cell level. The union of Human Protein Reference Database, STRING database (confidence scores > 600) and the human functional protein interaction network constructed by Wu et al.^37–39^ were combined as a network template, and edges were weighted by expression correlation (|PCC|) for each stage transition across all data points in the cpRNA-seq before and after the transition. We determined the optimal gamma in eResponseNet based on the trade-off between co-citation index (CI) of the genes in the selected eResponseNet with the keyword “neuron”^40^ and the smallest edge weight (|PCC| > 0.6) in the network.

### Detecting activation of signaling pathways

To determine the activation signaling pathways at each differentiation stage, we require that both the genes in a pathway and the upregulated targets of the pathway are enriched in a stage-specific highly expressed gene set. Here stage-specific highly expressed genes are defined as differentially expressed genes between one stage and the rest using RankProd in R (pfp < 0.05). The signaling pathways’ genes were collected from KEGG, GO, or Wiki pathway database (following that order). Fisher’s exact test was used to examine the significance of enrichment (Bonferroni-corrected *p*-value < 0.05). To obtain the upregulated target genes, we collected seven GEO datasets (GSE48092, GSE41260, GSE42565, GSE38719, GSE15268, GSE23239, and GSE31544) on the perturbation of seven development-related signaling pathways (BMP, FGF, Hedgehog, JAK-STAT, Notch, Nodal, and Wnt) and identified differentially expressed genes using RankProd in R (pfp < 0.1). The significance of enrichment for upregulated targets of a pathway over the rank of average gene expressions for each stage after *Z*-score normalization was determined using GSEA (FDR < 0.05).

### Construction of CSI network

The signaling pathway genes enriched in the eResponseNet, together with cell-cycle checkpoint genes were used to construct a CSI network (cutoff: CSI > 0.6) for each stage transition. The absolute pairwise PCC of these genes across the stages before or after the transition was used to calculate a CSI score according to ref.^23^. Then the three networks are visualized together in one CSI network, with node color denoting the stage in which signaling pathway nodes are activated.

### Construction of Cas9 and sgRNA expression plasmids

The expression vector px330-mcherry was used to express Cas9 and two sgRNAs. Guide sequence was designed according to the sequence of mouse *Fyn* gene (using the tool at http://crispr.mit.edu/). Oligonucleotides of sgRNA of upstream and downstream (fyn-sg-up-F: 5′- AGGCCCCTCAGGATTCGGAT-3′, fyn-sg-up-R: 5′-ATCCGAATCCTGAGGGGCCT-3′; fyn-sg-down-F: 5′-CCGACATGCAACCGAGCACT-3′, fyn-sg-down-R: 5′-AGTGCTCGGTTGCATGTCGG-3′) were annealed and inserted into separate pX330-mCherry vectors at the *Bbs*I site.

Plasmids of upstream and downstream were transfected to the E14 mESCs (originally from Austin Smith’s laboratory) together using Lipofectamine 2000. Genomic DNA from FACS sorted clonal RFP-cells was screened for specific site disruption by PCR amplification and sequencing (fyn-ko-PCR-F: 5′-TGGGTTTGGTACAGAGAGAAAG-3′, fyn-ko-PCR-R1: 5′-AGCACTTGACGCTGCTAAT-3′, fyn-ko-PCR-R2: 5′-GGGTCAAGGGTGTTAACCATAG-3′). The KO clone we used in this study harbors a 24,209 bp nucleotide deletion on chromosome 10 from coordinates 39,511,090 to 39,535,298 corresponding to amino acids 1 to 373, resulting in a complete lack of protein-level expression due to the deletion of the initiation codon.

### Data availability

The authors declare that all data supporting the findings of this study are available within the article and its supplementary information files or from the corresponding author on reasonable request. The bulk and single-cell RNA-seq data reported in this paper have been deposited in NCBI GEO under accession code GSE85234. The iCpSc package can be downloaded from http://www.picb.ac.cn/hanlab/iCpSc.html.

## Electronic supplementary material

Supplementary Information

Description of Additional Supplementary Files

Supplementary Data 1

Supplementary Data 2

Supplementary Data 3
