# Supplementary material for: Inference of differentiation time for single cell transcriptomes using cell population reference data
Source: Nat Commun. 2017 Nov 30;8:1856. doi: 10.1038/s41467-017-01860-2 (PMC5707349; doi:10.1038/s41467-017-01860-2)
Supplement: Supplementary file 1 — Supplementary Information [file 41467_2017_1860_MOESM1_ESM.pdf]

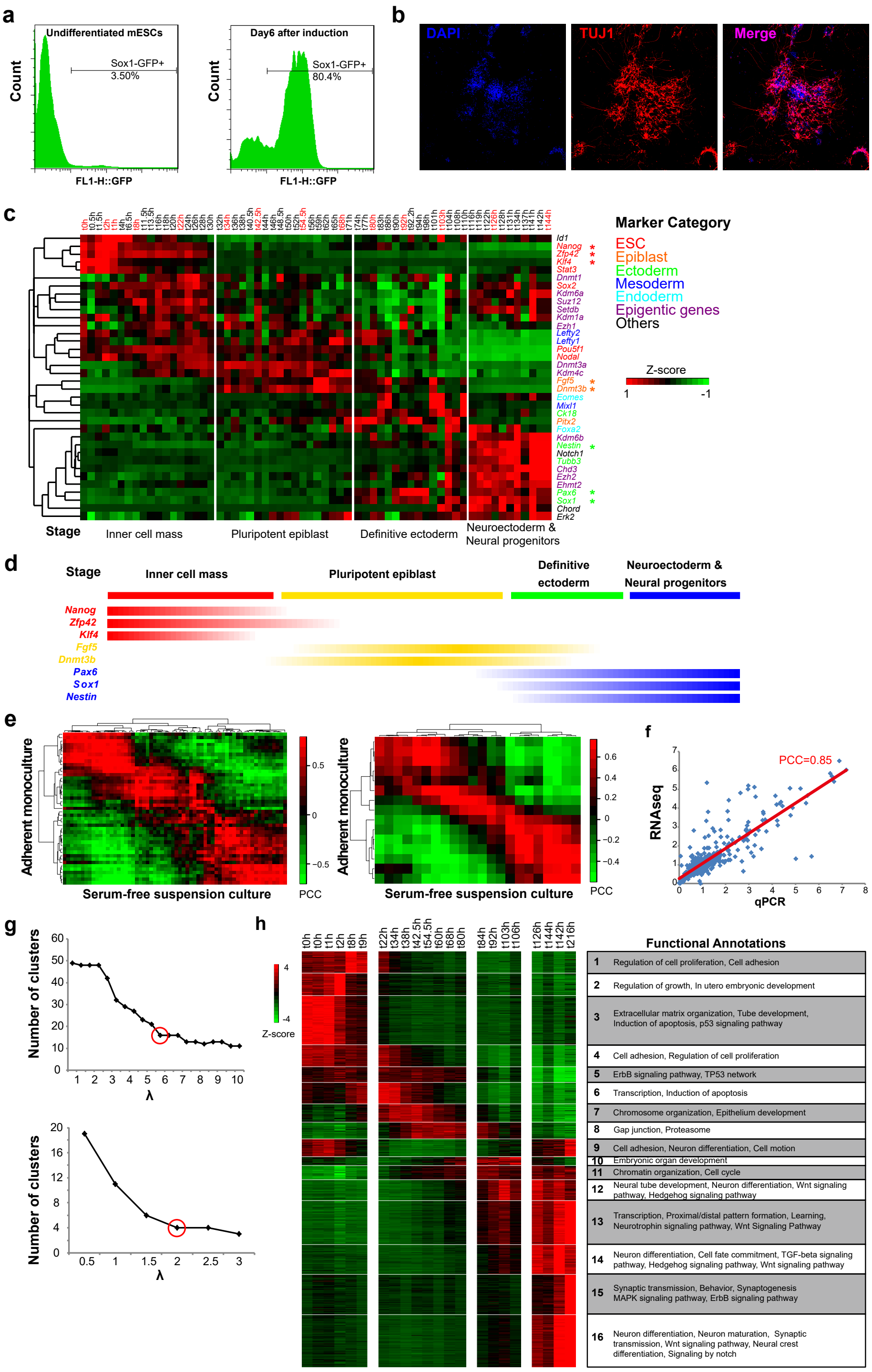

Supplementary Figure 1. Assessing the efficiency of neural differentiation of mouse embryonic stem cells (mESCs) and inter-cell heterogeneity revealed by single-cell RNA-seq

(a) Flow cytometry profiles of Sox1-GFP fluorescence.

(b) Immunostaining of TUJ1 3 days after the cells were replated onto PDL/laminin/fibronectin coated dishes (Methods).

(c) Fluidigm qPCR gene expression profiles of marker genes representing pluripotency, epiblast, the three germ layers, epigenetic enzymes and neural differentiation. The genes marked with stars represent known markers for differentiation stages. The red-font time points were selected to perform cell population RNA-seq.

(d) Marker genes' expression patterns used to define major differentiation stages.

(e) Expression correlations across temporal marker genes measured by Fluidigm qPCR (left) or all detected genes by cell population RNA-seq (RPKM > 1 in at least 2 samples, right) at different time points during differentiation by two different methods, serum-free suspension culture (x-axis) and adherent monoculture (y-axis).

(f) Correlation between marker genes' expression levels measured by RNA-seq and qPCR at the different time points.

(g) The number of clusters under different lambda for cpDEGs and 22 differentiation time points using BIC-SKmeans is plotted, and the number of clusters at the turning point is selected.

(h) Differentially expressed genes between every two samples identified by Cuffdiff from the cell population RNA-seq data (cpDEGs) are grouped into 16 gene clusters and 4 sample clusters by BIC-SKmeans.

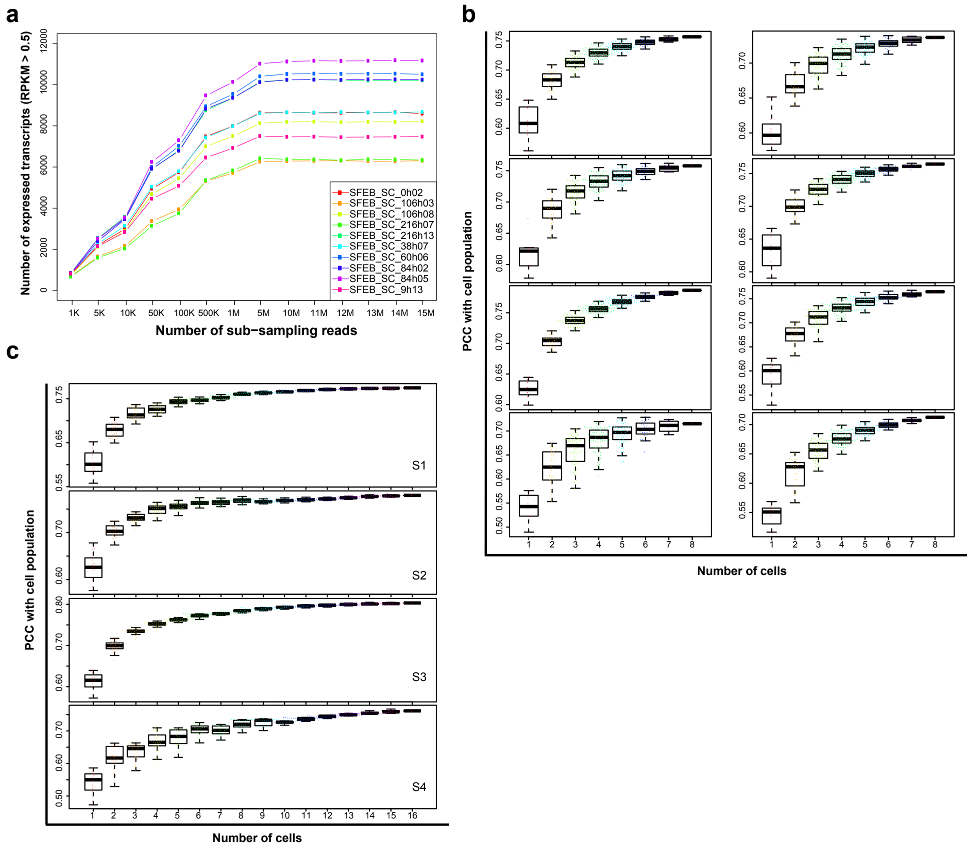

Supplementary Figure 2. Assessing the sampling saturation of scRNA-seq using cpRNA-seq as references

(a) Saturation analysis. X-axis is number of sub-sampling reads, and y-axis is number of transcripts with expression (RPKM > 0.5).

(b - c) scRNA-seq sampling saturation curves for each stage (j) or time point (j). In each panel, x-axis shows the number of randomly selected single cells, and y-axis shows the PCC of gene expression levels between cell population sample and averaged single cells based on all expressed genes.

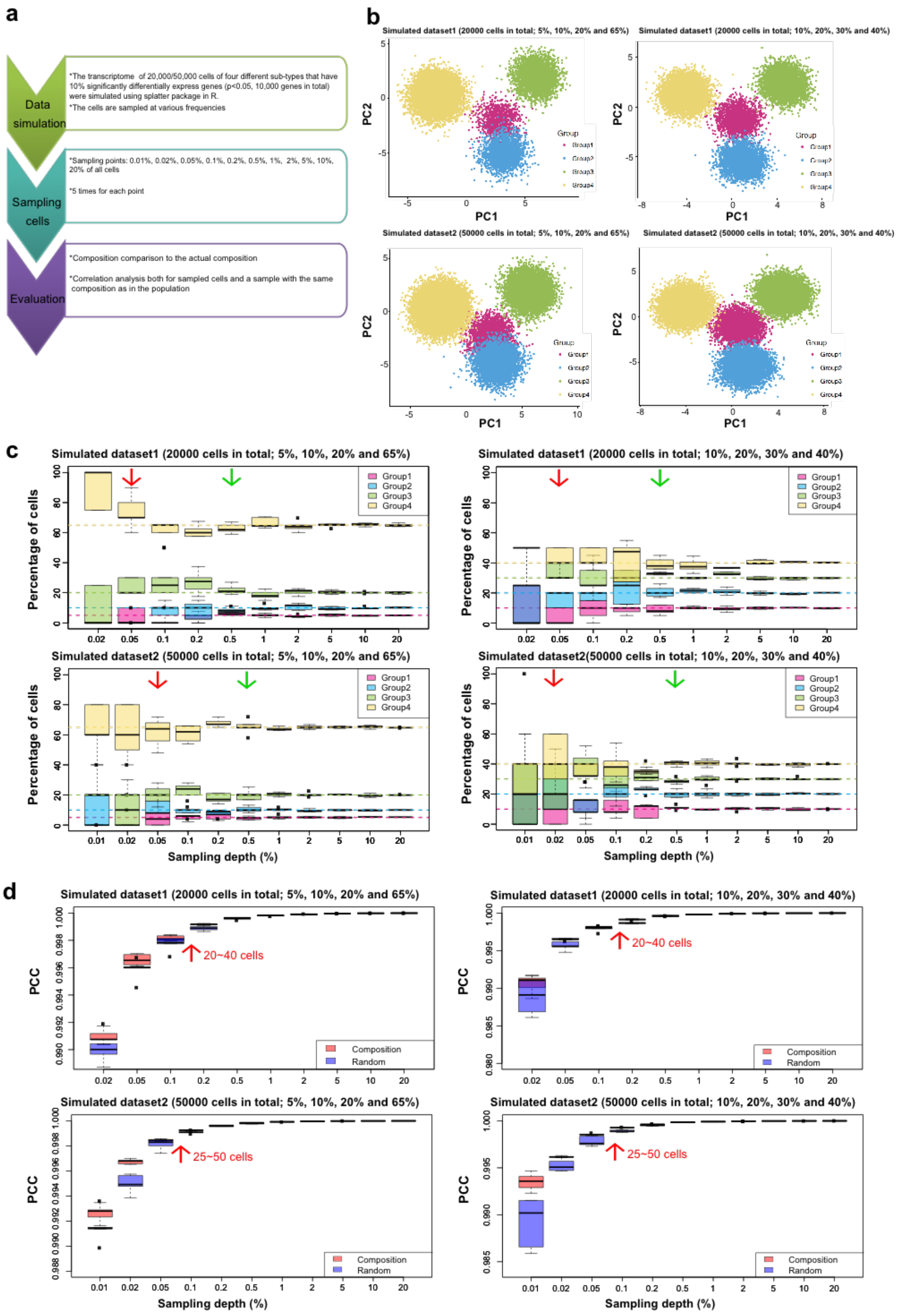

Supplementary Figure 3. Sampling of simulated data

(a) Workflow of simulation analysis.

(b) PCA based on the simulated single cells with 10% significantly differentially express in a total of 10,000 genes (p<0.05) using the splatter[1] software. Top: simulation with 20,000 cells; bottom: simulation with 50,000 cells; left: a cell population with four sub-types of 5%, 10%, 20% and 65%; right: a cell

population with four sub-types of 10%, 20%, 30% and 40%. The four colors represent four sub-types of cells.

(c) Composition analysis (top: simulation with 20,000 cells; bottom: simulation with 50,000 cells; left: a cell population with four sub-types of 5%, 10%, 20% and 65%; right: a cell population with four sub-types of 10%, 20%, 30% and 40%). The four colors represent four sub-types of cells, and the dash lines show the actual proportions of the subtypes in simulated data. Red arrows mark the starting points when all four cell types can be detected. Green arrows mark the starting points when the cell compositions become the same as the actual compositions.

(d) Correlation (measured by PCC) of the total transcriptome of sampled single cells to the original cell population. Top: simulation with 20,000 cells; bottom: simulation with 50,000 cells; left: a cell population with four sub-types of 5%, 10%, 20% and 65%; right: a cell population with four sub-types of 10%, 20%, 30% and 40%. Arrows mark the starting positions where similarities of single-cell to cell-population data start to plateau, and become similar between composition-fixed sampling and random sampling.

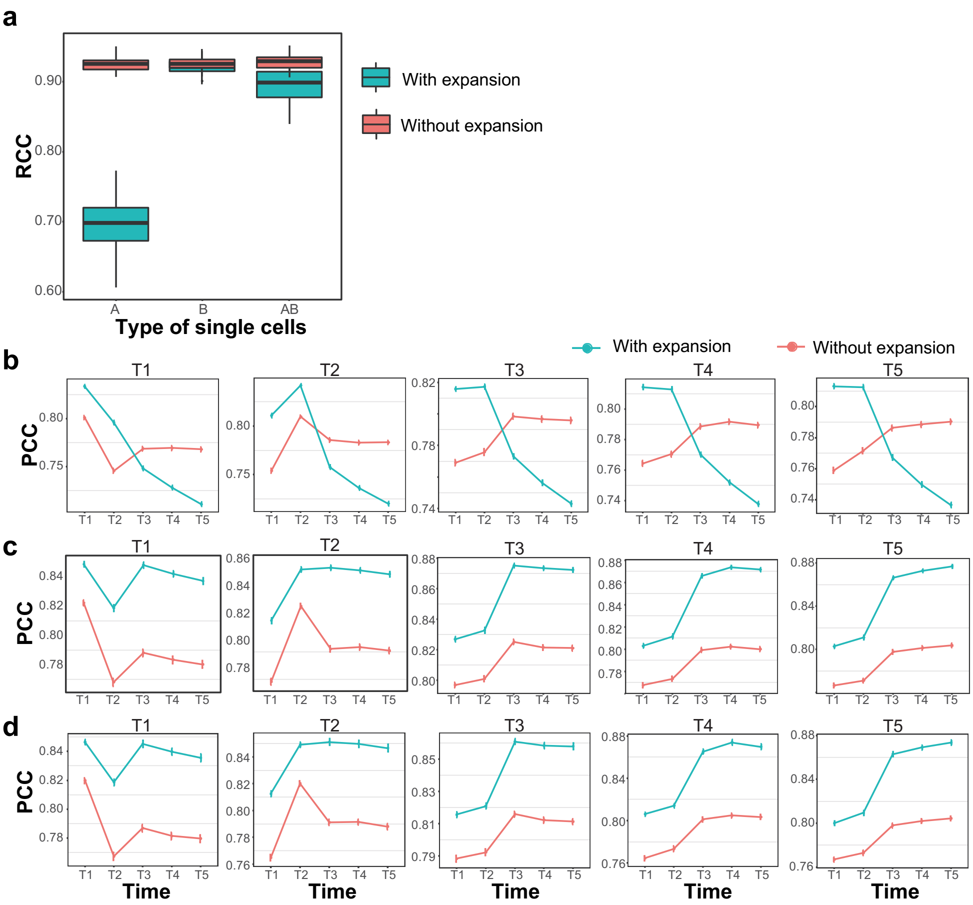

Supplementary Figure 4. Comparison of temporal trajectory mapping by modeling differentiation with or without unbalanced cell population expansion

A type and B type of cells have 20% of 10,000 genes differentially expressed ($p<0.05$, generated using the splatter[1] package). Then the two sub-populations of single cells change at each timepoint, a total of 5 timepoints with 1% of genes change significantly at each timepoint ($p<0.05$, generated using the splatter[1] package). From the starting time point T1 with 50% of A type of cells and 50% B type of cells, A and B cells either remain at the constant 50% throughout T1 to T5 (without expansion), or B cells expand linearly to 95% at T5 (with expansion). (a) Spearman's correlation coefficient (RCCs) between the true order of single cell and the order predicted by iCpSc.CpToScTime package. (b-d) PCC of all single cells of A (panel b), B (panel c) and A and B types combined (panel d) to the simulated cell population profiles at each time point in the no expansion model and the expansion model, respectively.

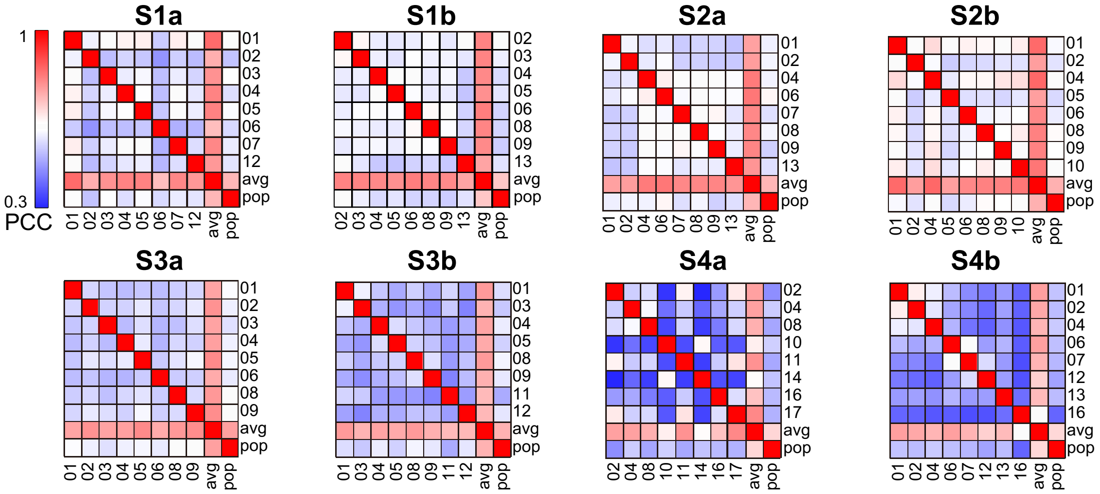

Supplementary Figure 5. Global correlations in gene expression between two single cells, or between the single cell average and cell population at each time point during mESC neural differentiation

Shown are the Pearson correlation coefficients (PCCs) between global expression profiles across all expressed genes (RPKM > 0.5 in at least eight samples). All correlations were calculated on log-transformed expression RPKM.

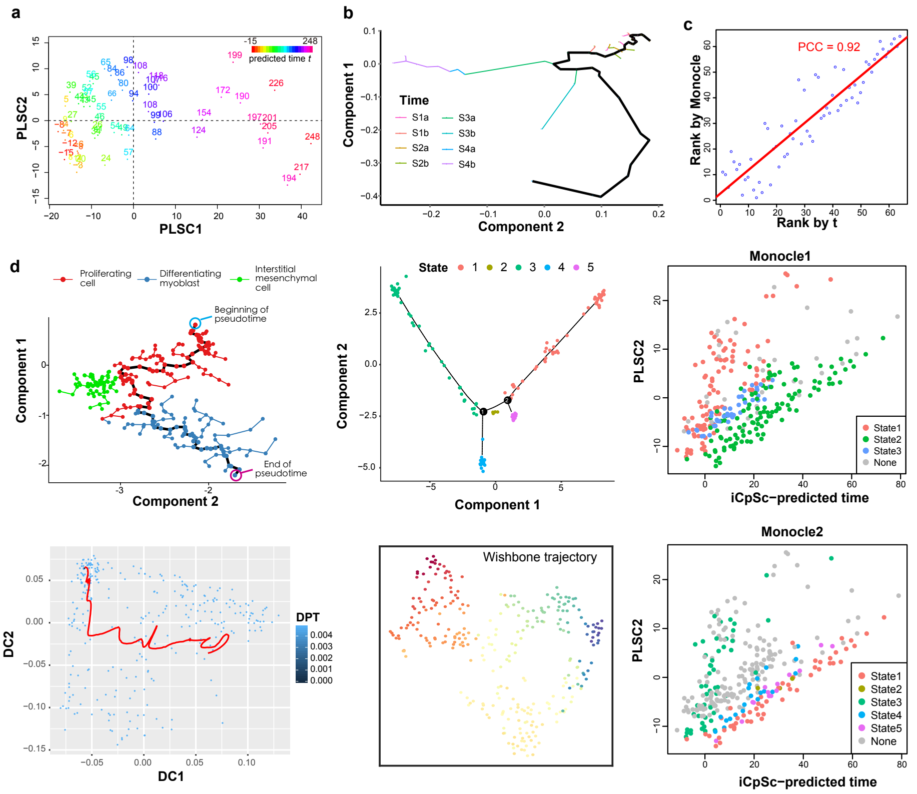

Supplementary Figure 6. Ordering single cells by predicted differentiation time *t*

(a) Temporal order displayed by single cells when projected on the first and second Partial Least Square (PLS) regression components of single cell RNA-seq profiles. The predicted differentiation time *t* of each single cell is shown.

(b) Prediction of differentiation trajectory by Monocle.

(c) The rank correlation of single cell orders given by PLSC1, 2 based predicted differentiation time *t* (or by SVR) with orders given by Monocle.

(d) Branching trajectories detected by the four (pseudo-) time prediction methods. Upper panels, trajectories and states detected by Monocle1 and 2. Middle panels, iCpSc-predicted time against PLSC2 displays three branches that diverge as time proceeds, with cells colored by the Monocle1 and 2 states, respectively. Lower panels, the trajectory identified by DPT with no clear branching detected (left) and the trajectory identified by Wishbone (right).

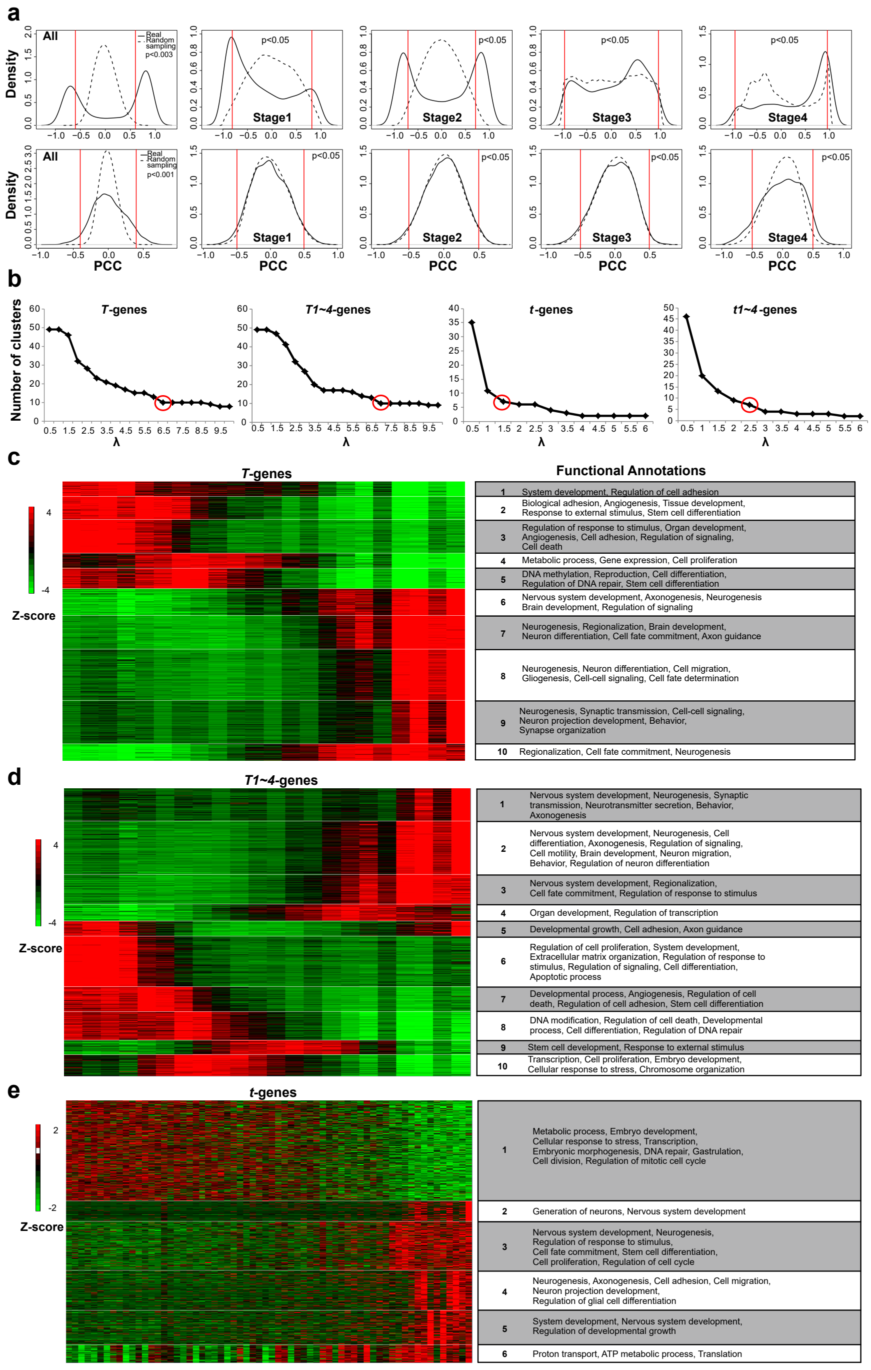

Supplementary Figure 7. Gene expression associated with differentiation time

(a) Distributions of gene expression PCCs to the fitted or predicted differentiation time *T* or *t* in real data or shuffled data across all samples or

stage-specific samples. Solid lines stand for real data of all genes, and dashed lines stand for the distribution based on 1000 times of sample randomization. Red vertical lines mark the positions of PCC cutoffs used for each data set.

(b) Searching for the optimum number of clusters to identify the expression patterns of *T*-genes and *T1~4*-genes across cell population time points, *t*-genes and *t1~4*-genes across single cells. The number of clusters under different lambda in BIC-SKmeans is plotted, and the number of clusters at the turning point is selected.

(c - e) The expression patterns of *T*- or *T1~4*- or *t-genes* across fitted differentiation time *T* in cell population or predicted differentiation time *t* in single cell, and the representative enriched functional terms for each cluster.

a

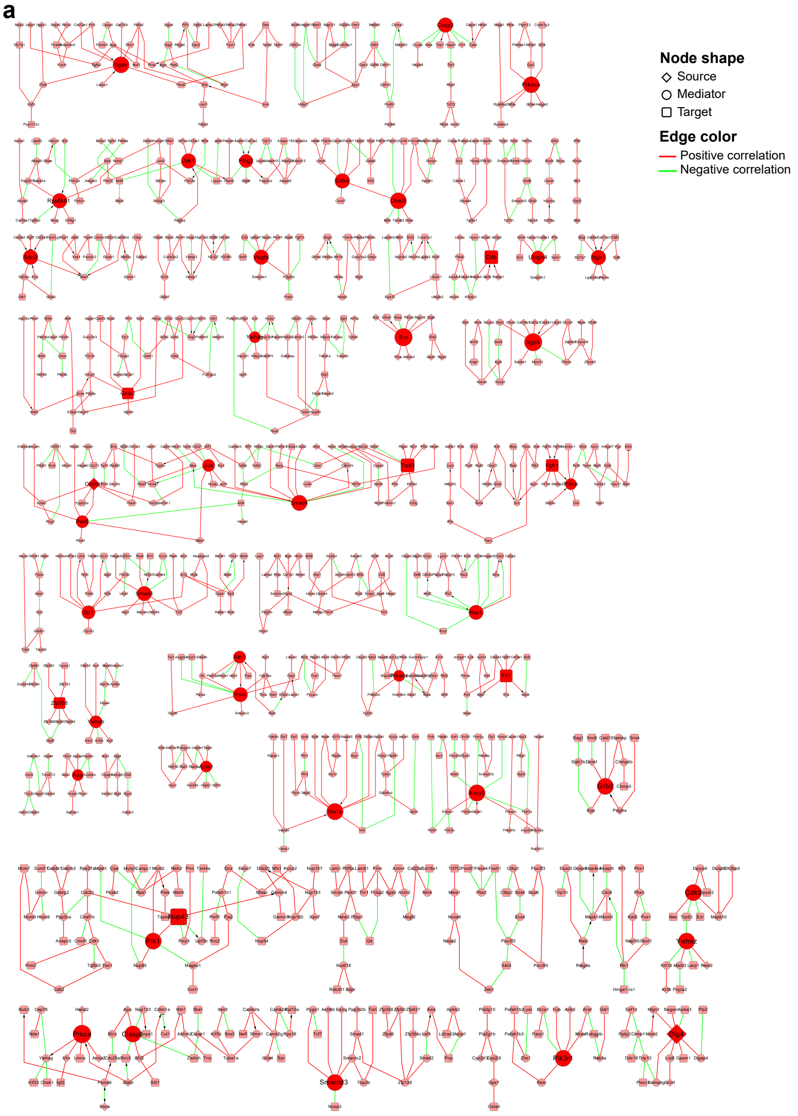

b

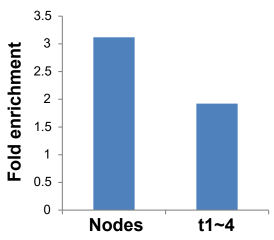

c

	BMP	FGFR	Hedgehog	JAK-STAT	Nodal	Notch	Wnt
Stage1	0.501	1.621	-0.822	1.796	0.038	-0.808	-0.992
Stage2	-1.025	-0.349	-0.963	-0.646	1.332	-1.373	-1.016
Stage3	1.48	0.333	3.4	-1.296	-0.159	-0.77	1.166
Stage4	0.493	0.344	2.146	-1.478	-1.244	3.277	3.732

Supplementary Figure 8. Inferring a regulatory network for mESC neural differentiation timing by eResponseNet and CSI networks

(a) All three stages transition eResponseNets' components that contain hub genes. Only hubs and their first-degree interactors are included in the networks.

(b). Fold enrichment of cell cycle genes in eResponseNet nodes and

t1~4-genes, respectively.

(c) Significance of association of 7 development-related signaling pathways genes with the stage specifically highly expressed genes in each differentiation stage. Significance was determined by Fisher's exact test. -log10 transformed Bonferroni corrected p-values are shown, and highlighted red when p-value < 0.05.

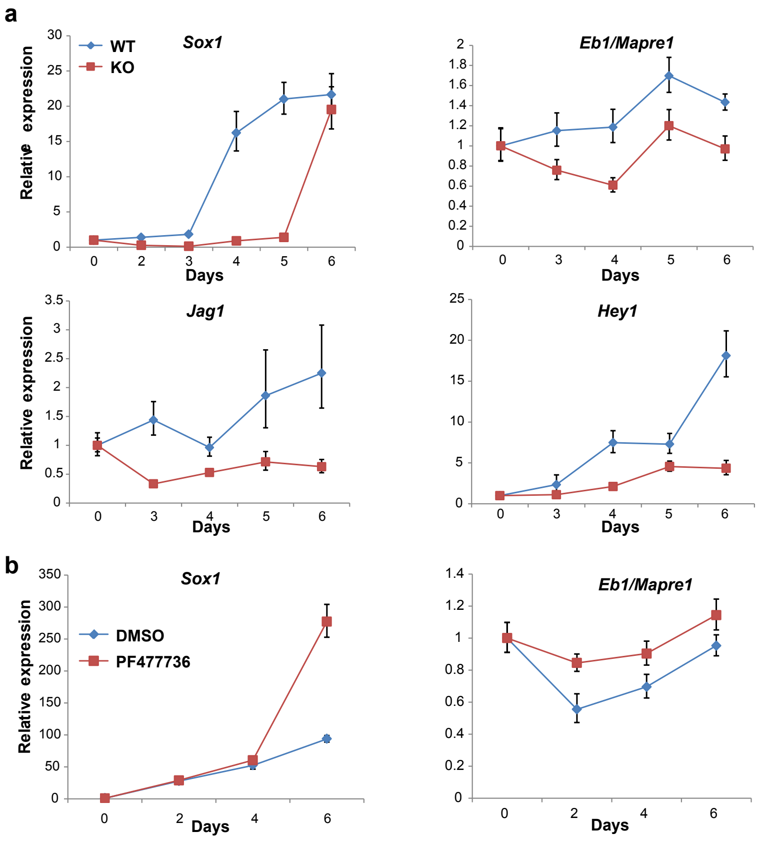

Supplementary Figure 9. Regulation of neural differentiation timing and cell cycle by Fyn (replicate experiments for Fig. 4)

(a) Biological replicate for Fig. 4a.

(b) Biological replicate for Fig. 4c.

Supplementary Table 1. Real-time PCR primers

Gene	Sequence of Forward Primer	Sequence of Reverse Primer
Gapdh	GCCTTCCGTGTTCCTACC	CCTCAGTGTAGCCCAAGATG
Actb	ACCTTCTACAATGAGCTGCG	CTGGATGGCTACGTACATGG
Zfp42	ACATCCTAACCCACGCAAAG	CATTAAGACTACCCAGCCTGAG
Nodal	CCAACCATGCCTACATCCAG	CTGCCATTGTCCACATAAAGC
Fgf5	AGAGTGGGCATCGGTTTC	CTCGTATTCCTACAATCCCCTG
Sox2	CACATGGCCCAGCACTAC	CCCTCCCAATTCCCTTGTATC
Sox1	CTTCATGGTGTGGTCCCG	TTGCTGATCTCCGAGTTGTG
Chord	AGTTCTATTGGCTGGGCTTG	ATCTTTTACCACGCCCTGAG
Krt18	ACACCAACATCACAAGGCTG	TTCCACAGTCAATCCAGAGC
Erk2	CTACACCAACCTCTCGTACATC	CTCTTAGGGTTCTTTGACAGTAGG
Eomes	CGGCACCAAACTGAGATGA	CAGAACCACTTCCACGAAAAC
Klf4	ACTTGTGACTATGCAGGCTG	ACAGTGGTAAGGTTTCTCGC
Tubb3	CGCCTTTGGACACCTATTCAG	TTCTCACACTCTTTCCGCAC
Notch1	ATGTCAATGTTCGAGGACCAG	TCACTGTTGCCTGTCTCAAG
Foxa2	CGGGACTTAACTGTAACGGG	TCATGTTGCTCACGGAAGAG
Pitx2	CAATCTCCGATACTTCCAGCC	CCCACATCCTCATTCTTTCCT
Pax6	CCCTCACCAACACGTACAG	TCATAACTCCGCCCATTCAC
Nes	AAGTTCCCAGGCTTCTCTTG	GTCTCAAGGGTATTAGGCAAGG
Mixl1	TTTCTGCCTTAGGTAACTTCTGAG	CAGTGTCAGAGAAGGGAACC
Id1	ATGTGTTCCAGCCGACG	GGTAGTGTCTTTCCCAGAGATC
Lefty1	CACAAGTTGGTTCGTTTCGC	CATCGGGTGCCTTCAGTC
Lefty2	CGTGAGGTCCCAGTATGTG	CTCCATTCCGAACACTAGCAG
Ehmt2	ACCATGTCCAAACCTAGCAAC	CTACCAGAGTTCAGCTTCCTC
Setdb1	GGCTATGCTTCCTATGTCACTC	ATTGGGCGGTTTGGATAGG
Kdm4c	CCCTGTGTATCGTACTTTCCTG	CTGTCCCTTTGTCTTCCCTC
Kdm1a	AGCTACAGAGTTTTGGGATGG	GATTCCCTCCAAGACCTGTTAC
Ezh2	TCCCGTTAAAGACCCTGAATG	TGAAAGTGCCATCCTGATCC
Ezh1	TCTTCCACGGCACCTATTTC	TTCTGTGCAGGGTTCATGAG
Kdm6b	GTCCACTTCCAACTCCATCTG	AACCAATTCCAGCCTCCG
Chd3	GTCCTTGTACCTCTAAGCCTG	CCTCTGCCTCCATCTTTTCC
Pou5f1	AGTGGAAAGCAACTCAGAGG	AACTGTTCTAGCTCCTTCTGC
Kdm6a	GTGGAAGTAATGGAAACGTGC	TGTGAACTCGGACCTTTGTG
Dnmt3b	GTACCCCATCAGTTGACTTGAG	TTGATCTTTCCCCACACGAG
Dnmt3a	GGACTTTATGAGGGTACTGGC	GATGTCCCTCTTGTCACTAACG
Dnmt1	GACCTACTTGAGAGCATCCAG	TTCCCTTTCCCTTTGTTCCC
Stat3	GGCACCTTGGATTGAGAGTC	CGAAGGTTGTGCTGATAGAGG
Nanog	CAGAAAAACCAGTGGTTGAAGACTAG	GCAATGGATGCTGGGATACTC
Suz12	CTCAGGATATACATCGCCAACC	GCTCCACTTCTCCATCTTCAG

Supplementary Table 2. Fold enrichment of signaling genes in source,

target and eResponseNet nodes

Gene set	Fold enriched
Transition1.source	1.778932795
Transition2.source	1.663701008
Transition3.source	1.786104576
Transition1.target	1.289667682
Transition2.target	1.195727268
Transition3.target	1.025077796
Transition1.eResponseNet_nodes	4.590911215
Transition2.eResponseNet_nodes	4.087956471
Transition3.eResponseNet_nodes	4.478995058

Supplementary Table 3. Significance of interactions between a cell cycle checkpoint and a signaling pathway in the eResponseNet networks (1000 permutations)

Cell cycle checkpoint	Signaling pathway	p-value
G1S	JAK-STAT	0
G1S	Wnt	0
G1S	Nodal	0.003
G1S	Hedgehog	0.011
G1S	Notch	0.016
G1S	BMP	0.034
G1S	FGF	0.053
G2M	JAK-STAT	0
G2M	Nodal	0.001
G2M	Hedgehog	0.005
G2M	BMP	0.021
G2M	Wnt	0.051
G2M	FGF	0.111
G2M	Notch	0.169
MG1	Hedgehog	0
MG1	Notch	0
MG1	BMP	0.048
MG1	Wnt	0.268
MG1	Nodal	0.391
MG1	JAK-STAT	0.468
MG1	FGF	0.58

Supplementary Table 4. Hub genes in the eResponseNets and CSI

networks

Network	Gene symbols
eResponseNet	*Rhog, Traf6, Smad4, Adcy5, Itgb5, Rac1, Rxra, Smad3, Src, Stx1a, Dlg4, Fyn, Hdac2, Itgav, Prkaca, Smad1, Sp1, Trp53, Ube2i, Akt1, Casp3, Cdk4, Cdkn1a, Ctnnb1, Ephb2, Fgfr1, Fn1, Gsk3b, Itgb1, Jak1, Jun, Junb, Mmp2, Pax6, Pik3cb, Pik3r3, Prkca, Rps6kb1, Sdc2, Thbs1, Vegfa, Ywhab, Acta1, App, Cdk5, Clasp2, Cltb, Dvl3, Gnai2, Grb2, Nup43, Pik3r1, Plcg2, Plk1, Prkce, Smarcd3, Tnfrsf1a, Ubqln4, Ywhag, Ywhaz, Zfp358*
CSI	*Birc5, Chek2, Dtx3, Ascl1, Myh6, Stk36, Hey1, Bmpr1a, Dvl3, Fgf8, Gsk3b, Dll3, Bmp7, Haus4, Dll1, Fyn, Stmn1, Foxh1, Gdf1, Id1, Msx2, Mfng, Apc, Adam10*

Hubs are defined as the nodes (genes) with the degree k greater than 4 for eResponseNets and 20 for the CSI network corresponding to top 5% and 20% k in the networks, respectively.

Supplementary References

1 Zappia, L., Phipson, B. & Oshlack, A. Splatter: Simulation Of Single-Cell RNA Sequencing Data. *bioRxiv*, 133173 (2017).
