# Supplementary material for: Inference of differentiation time for single cell transcriptomes using cell population reference data
Source: Nat Commun. 2017 Nov 30;8:1856. doi: 10.1038/s41467-017-01860-2 (PMC5707349; doi:10.1038/s41467-017-01860-2)
Supplement: Supplementary file 2 — Description of Additional Supplementary Files [file 41467_2017_1860_MOESM2_ESM.pdf]

Description of Additional Supplementary Files

File Name: Supplementary Data 1
Description: Functional annotations for clusters of cpDEGs,

File Name: Supplementary Data 2
Description: Fitted or predicted differentiation time T or t related genes sets,

File Name: Supplementary Data 3
Description: Functional annotations for clusters of t1~4-genes
